# Supplementary material for: ROR1 as an Immunotherapeutic Target for Inducing Antitumor Helper T Cell Responses Against Head and Neck Squamous Cell Carcinoma
Source: Cancers (Basel). 2025 Jul 12;17(14):2326. doi: 10.3390/cancers17142326 (PMC12293936; doi:10.3390/cancers17142326)
Supplement: Supplementary file 1 [file cancers-17-02326-s001.zip › cancers-3719857-supplementary.pdf]

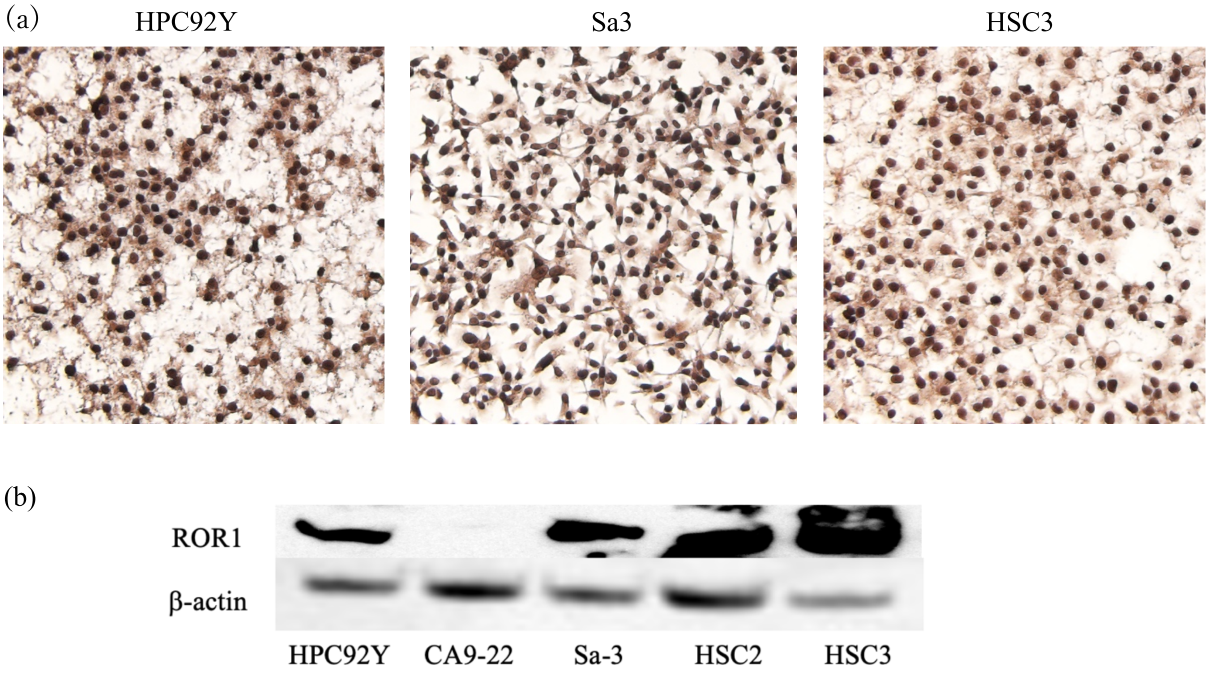

Supplemental Figure S1. ROR1 expression in the HNSCC cell lines HPC92Y, Sa3, and HSC3.

(a) Representative immunohistochemical images of ROR1 expression in tumor cells.

(b) ROR1 and β-actin western blotting images in tumor cells. Polyclonal rabbit anti-human ROR1 Ab (1:250; Santa Cruz Biotechnology, Santa Cruz, CA) and monoclonal mouse anti-β-actin Ab (C4, 1:1000; Santa Cruz Biotechnology, Santa Cruz, CA) were used.

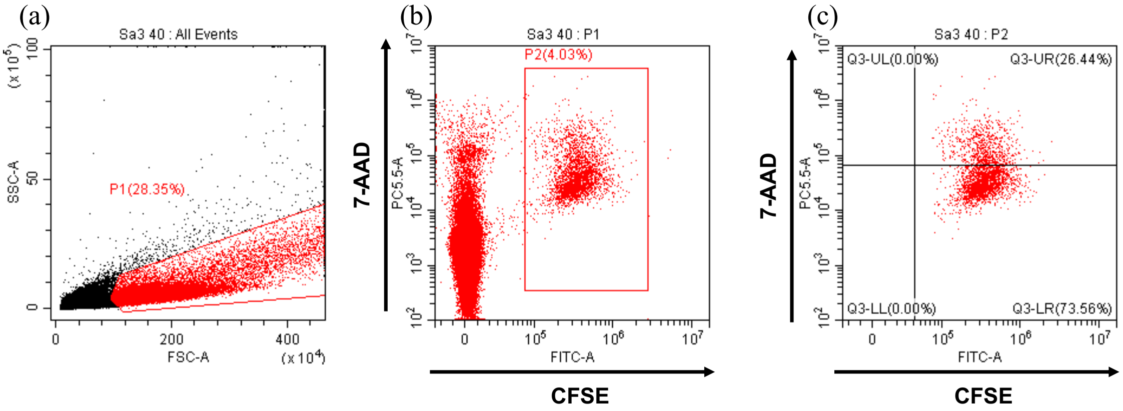

Supplemental Figure S2. The representative examples of flow gating of cytotoxicity assay.

(a) Debris exclusion. (b) Identification of CFSE+ tumor cells. (c) Identification of 7-AAD+ dead tumor cells (upper right) and live tumor cells (lower right).

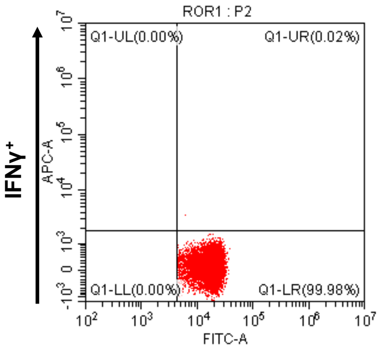

Supplemental Figure S3. Detection of IFN-γ–producing CD4^{+} T cells by flow cytometry following short-term stimulation with the ROR1$_{403–417}$ peptide.

PBMCs obtained from healthy control 2 were stimulated overnight with the ROR1$_{403–417}$ peptide. Among CD4^{+} T cells, intracellular staining for IFN-γ revealed that only 0.02% (upper right) of CD4^{+} T cells produced IFN-γ in the presence of ROR1$_{403–417}$ peptide.
